# Informed Consent in Patients Undergoing Primary Hip and Knee Arthroplasty: What Do Patients Want to Know?

**DOI:** 10.7759/cureus.8457

**Published:** 2020-06-05

**Authors:** Nemandra A Sandiford, Maalee Mahendra, Lilanthi Wickramarachchi, Diane Back, Mohit Bansal

**Affiliations:** 1 Joint Reconstruction Unit (Hip and Knee), Southland Teaching Hospital, Invercargill, NZL; 2 Orthopaedic Surgery, Guy's and St Thomas' Hospital, London, GBR; 3 Trauma and Orthopaedics, Guy's and St Thomas' Hospital, London, GBR

**Keywords:** informed consent, arthroplasty, replacement, standards, patient focus

## Abstract

Introduction

The consenting process has been surgeon-focussed traditionally, but there is a recent trend towards making the process more patient and procedure-focussed. The primary aims were to identify the risks considered most important and requiring further discussion by the patients undergoing primary total hip arthroplasty (THA) and primary total knee arthroplasty (TKA), as well as to identify the sporting and recreational activities these patients would like to pursue after surgery according to the age group, taking into consideration their values and expectations. The secondary aim is to assess the compliance of the current consenting process with guidelines set out by a governing body in a tertiary referral arthroplasty unit.

Material and method

A prospective study reviewing the consenting process was carried out on 137 patients undergoing THA or TKA over a 12-month period in a tertiary teaching hospital. Patients unable to complete a questionnaire and undergoing revision or uni-compartment arthroplasty were excluded. A standardized anonymous questionnaire was administered. Patients were asked to fill in the specific activities they considered important to be discussed. The data were tabulated in Microsoft Excel (Microsoft Corporation, Redmond, Washington) and subgroup analysis was performed using the student's t-test. The level of statistical significance was p=0.05. Two-hundred consent forms were reviewed to assess whether the information entered correlated to the guidelines presented in Ortho-Consent.

Results

One-hundred thirty-seven questionnaires were reviewed. The mean age was 66 (range 45-91), with the majority of patients undergoing TKA (114) versus THA (23). The patients in active employment were more concerned about blood clots, pain, joint failure, limb length discrepancy, and infection. Patients undergoing TKA wanted more information on pain management and joint longevity, which achieved statistical significance. There was a significant difference in the activities patients would like to pursue as well as in expectations amongst different age groups. The quality of documentation in the consent form was quite variable in discussing complications, surgery benefits, and alternative treatments.

Conclusion

Obtaining consent is a patient-specific process. Patient perception of important points that merit discussion can vary with age and employment status. Return to driving is important for all ages, however, as the population ages, the ability to return to activities of daily living becomes an increasingly important discussion point during the consent process.

## Introduction

Consent remains one of the fundamental elements of the patient-surgeon relationship. The task of obtaining informed consent from patients prior to their proposed operation forms a major part of a surgeon’s workload and is a key element of good medical practice [1]. Consent to treatment is based on the principle that a person must give informed permission before they receive any type of medical treatment, test, or examination, with a full understanding of the benefits, risks, and alternative treatments. In common orthopedic practice, the risks that are considered ‘reasonable’ to discuss are those with an incidence of 1% or greater [1]. The practice of obtaining consent is variable. The grade of the doctor, timing, location, and delivery of consent varies considerably between hospitals and is often performed by junior doctors who lack the experience required to discuss the specific details and risks of the procedure.

Pre-printed and pre-filled consent forms are used in several units. One such system is Ortho-Consent, which is supported by some governing bodies such as the British Orthopaedic Association (BOA) (Annexures 1 and 2) [2]. From a medico-legal perspective, consent is considered to be appropriate if deemed to be so by a group of peers (the Bolam test) [3].

Traditionally, the consenting process has been focussed around issues considered pertinent and important by the surgeon. The recent case of Montgomery vs Lanarkshire has led to suggestions for a significant change in the consenting process for patients undergoing total joint arthroplasty [4]. The aim is to make the consenting process more patient-specific and represents a significant change from current practice. Several authors have examined the methods of obtaining appropriate consent but there is a paucity of data on what aspects of total joint arthroplasty patients themselves consider important and how this compares to current practice. An understanding of patient-specific issues is therefore important. To our knowledge, there has been no previous study comparing the issues considered important by surgeons and patients during the consent process.

The primary aims of the study are as follows:

Identify the risks considered most important and requiring further discussion by the patients undergoing primary total hip arthroplasty (THA) and primary total knee arthroplasty (TKA) according to the type of procedure and employment status.

Identify the sporting and recreational activities these patients would like to pursue after surgery according to the age group, taking into consideration their values and expectations.

The secondary aims are to assess the compliance of the current consenting process with guidelines set out by a governing body in a tertiary referral arthroplasty unit.

## Materials and methods

A prospective study reviewing the consenting process was performed between June 2015 and June 2016; 137 patients undergoing primary THA or TKA were included. Patients who were unable to complete their own consent forms and those undergoing revision arthroplasty, hemiarthroplasty, or uni-compartmental knee arthroplasty, as well as those who were unable to speak English, were excluded. All data were anonymized and collected by a research nurse. To assess our primary aim, a standardized anonymous questionnaire was administered to patients included in the study. Demographic data, occupation, type of arthroplasty performed, and what patients considered important information, points they wished to have addressed pre-operatively as well as specific activities that were important to them, were collected. Patients were also encouraged to enter issues not mentioned in the questionnaire, which they considered important, as free text (Annexure 3).

In order to assess our secondary objective, 200 consecutive consent forms were reviewed. The location and timing of consent, the person undertaking the consent, and the content of the discussion were compared to guidelines presented in the Ortho-Consent document, the details of which were based on legal and ethical advice and the guidelines from the Department of Health established following the Bristol Royal Infirmary Inquiry in 2001. The common risks identified were blood clots, bleeding, pain, prosthesis wear/loosening, limb length discrepancy, and joint dislocation (specific for hip replacement). Less common or rare complications included were infection, wound healing problems, nerve damage, bone damage, vessel damage, pulmonary embolism, and death. Data were tabulated using Microsoft Excel (Microsoft Corporation, Redmond, Washington). Statistical analysis was performed using the student's t-test. The level of statistical significance was p=0.05.

## Results

Complications following surgery

A total of 137 responses were analyzed. The mean age of our cohort was 66 years (range 45-91 years); 73 were males and 64 females. One-hundred fourteen (83%) patients underwent TKA and 23 patients (17%) THA. Seventy-four (54%) patients were employed and 63 (46%) patients were unemployed (or retired) at the time of completion of the questionnaire (Table 1).

**Table 1:** Patient demographics

Age group	Male	Female	Employed	Unemployed
41-50	3	3	4	2
51-60	18	19	27	10
61-70	35	21	18	38
71-80	13	16	5	24
81-90	4	5	0	9

Major complications for which more detailed information and discussion expected by the patients across all groups is presented in Table 2. The cohort of patients in active employment was more concerned about the risk of deep venous thrombosis (DVT, blood clots), postoperative pain, the durability of the prosthesis, limb length discrepancy, and infection when compared to patients not in employment. This did not reach statistical significance; however, the trend was clear. Ninety-two percent of patients who were in active employment wished to have major complications discussed as compared to 70% of those who were not in employment (p=0.74) (Table 2).

**Table 2:** Comparison of major complications considered important among employed and unemployed patients

Complications	Unemployed patients (N=74)	Employed or retired patients (n=63)	p-value
Blood Clots	12 (19%)	31 (42%)	p=0.74
Pain	50 (79%)	58 (92%)	p=0.91
Bleeding	27 (43%)	34 (46%)	p=0.95
Prosthesis wear	20 (32%)	45 (61%)	p=0.83
Life span of joint	54 (88%)	56 (89%)	p=0.71
Limb length discrepancy	22 (35%)	46 (62%)	p=0.83
Infection	28 (44%)	46 (62%)	p=0.90

Patients undergoing THA and TKA were assessed separately. Similar numbers of patients in each cohort were concerned about DVT, bleeding, prosthesis wear, limb length discrepancy, and infection. A significantly larger number of patients undergoing TKA were more concerned about post-operative pain and the life span of the prosthesis (p=0.005 and p=0.008, respectively (Table 3).

**Table 3:** Comparison of major complications considered important among THA and TKA patients in percentage THA: total hip arthroplasty; TKA: total knee arthroplasty

Complications	THA (n=23)	TKA (n=114)	P-value
Blood clots	7 (30%)	36 (32%)	p=0.27
Pain	19 (83%)	102 (89%)	p=0.005
Bleeding	10 (43%)	51 (45%)	p=0.18
Prosthesis wear	12 (52%)	53 (46%)	p=0.17
Life span of joint	21 (91%)	99 (87%)	p=0.008
Limb length discrepancy	11 (48%)	57 (50%)	p=0.14
Infection	12 (52%)	61 (53%)	p=0.12

Sporting and recreational activity

One-hundred six (77%) patients requested a specific discussion on return to sporting and recreational activities during the consenting process (Table 4). Patients in the age group of 40-50 requested more information about playing racket sports and skiing. Driving was the main concern for patients between the ages of 50 and 70 (21/84, 32%) followed by swimming (16/84, 19%), gardening (14/84, 16.6%), hiking (10/84, 11.9%), and walking (9/84, 10.7%). Patients over the age of 70 requested a discussion on their ability to drive and the optimal time for doing this (5/22, 22.7%) following surgery, as well as their ability to return to activities like gardening (5/22, 22.7%) and household chores (5/22, 22.7%) (Table 5).

**Table 4:** Comparison of the difference in activities between age groups

Activities	<70 years (n=84) %	>70 years (n=22) %	P-value
Driving	32	23	p=0.31
Swimming	19	13	p=0.41
Gardening	17	23	p=0.49
Walking	11	14	p=0.51

**Table 5:** Important activities according to the age group

Age(years)	40-50	50-60	60-70	70-80	80-90
Activity	Running (2)	Driving (8)	Driving (11)	Driving (3)	Gardening (2)
	Driving (2)	Swimming (5)	Swimming (9)	Gardening (3)	Driving (2)
	Racket sports (2)	Hiking (5)	Gardening (9)	Swimming (3)	Walking to the shop (1)
	Swimming (2)	Gardening (5)	Walking (7)	Household activities (3)	Stairs (1)
	Cycling (1)	Walking (2)	Hiking (5)	Walking (2)	
	Skiing (1)	Yoga (2)	Dancing (3)	Golf (2)	
			Yoga (3)		

Patients also stated their desire to have several other issues discussed prior to surgery as part of the consent process. These included post-operative pain management, psychological effects of surgery, length of surgical procedure, operating surgeon’s name, implants to be used, time to get back to work fully, physiotherapy protocol, and ward contact details.

Compliance with consenting guidelines

The records of 200 patients who had undergone THA or TKA were compared to the guidelines outlined in the Ortho-Consent document. Alternatives to the operation were not documented in any of the consent forms. At least one benefit was documented in 100% of cases. The potential risks discussed and the frequency of these are presented in Table 6. The issues of prosthesis wear and death were discussed in significantly fewer cases than other complications (p=0.002). If these two factors are excluded, there was no difference in the frequency of the major and minor complications discussed (p=0.886).

**Table 6:** The frequency of major and minor complications as mentioned in the consent forms

Complications	Number of patients (%)
Major complications	
Blood clots (DVT)	173 (82.5)
Pain	115 (57.5)
Bleeding	158 (79)
Prosthesis Wear	23 (11.5)
Stiffness	101 (50.5)
Limb length discrepancy	124 (59)
Dislocations	140 (70)
Minor complications	
Infection	187 (93.5)
Scar	97 (48.5)
Nerve damage	177 (88.5)
Bone damage	134 (67)
Vessel damage	137 (68.5)
Pulmonary embolism	173 (86.5)
Death	11 (5.5)

## Discussion

Obtaining consent prior to surgical treatment is a fundamental part of good medical practice [1]. The ideal process for obtaining informed consent prior to surgery in patients undergoing THA or TKA is undecided. Issues relating to consenting are also an increasing source for medico-legal claims [2]. This is not only expensive for the treating institution and health service but also potentially reflects a degree of disparity between the issues considered important by the surgeon and those considered important and worthy of discussion by the patient. This was clearly exemplified by the recent case of Montgomery v Lanarkshire [3-5]. This case has led to a focus on the process of obtaining consent as well as the depth of information sharing with the patient. It challenges the Bolam test as a medico-legal defense as to the adequacy of the consent process and emphasizes the need to make it more patient-specific. The Montgomery case clarifies the consent law further and aligns it with the current General Medical Council (GMC) guidance [5]. Various studies have suggested that the patient literature available is quite variable and may not be fit for purpose to cover general and surgery specific information [6-9]. To the authors’ knowledge, this is the first prospective study looking specifically at patients' concerns and activity profiles depending on age and employment status around the consenting process for THA and TKA. There is little evidence that patients are consulted prior to or involved in the production of literature relating to THA [10]. An understanding of what patients really want to know in an informed consent procedure and addressing the concerns around the various aspects of daily life can improve the quality of this process and make it more individualized, patient-centered, and comprehensive [11].

In the United Kingdom, all the patients undergoing joint replacement surgery complete Patient-Reported Outcome Measures (PROMs) questionnaires relating to their health, quality of life, and effectiveness of the operation following surgery [12-13]. The aim is to use PROMs as a predictor of success following THA and TKA. This makes it imperative for the surgeons to have a robust consenting process addressing all the aspects of surgery that patients consider important and that will affect their quality of life. Our results suggest that issues considered important vary with age, level of function, and employment status. Although not statistically significant, patients in active employment are more concerned about factors that could affect their long-term function such as wear of the prosthesis, leg length discrepancy, scarring, infection, and returning to driving following surgery. Overall, there was no variation based on the type of operation, i.e. hip or knee replacement, but the patients undergoing TKA were more concerned about the post-operative pain and life span of the joint, which reached statistical significance [14-15]. This finding potentially reflects the widespread availability of information from sources such as the Internet, leaflets, and other patients who have had similar surgery.

The information from this study potentially can act as a guide and framework for patient-specific consent during total joint arthroplasty. Age influences the issues that patients consider important. Below the age of 70 years, patients are more concerned about returning to aerobic and fitness type activities, such as running, swimming, and hiking as well as household activities such as gardening. These were not addressed by the pre-filled printable consent forms commonly available online and reflect the importance of a bespoke, patient-specific consenting process. The definition of elderly is conventionally defined as age 65 years or older [16]. The results of this study suggest that this definition should not influence the treating clinician in providing information on return to activity and sports. A significant number of patients above the age of 65 years showed a desire to return to these activities following joint replacement and would like to discuss and gain more information on how and when to get back to them. The ability to maintain independence following surgery is important irrespective of age as reflected by the desire of patients across all age groups to have the issue of returning to driving discussed.

The use of online portals such as Ortho-Consent has been shown to improve the patients’ knowledge and access to information [17]. The results of this study have demonstrated considerable variation in the quality of the consenting process when compared to pre-printed formats. Major complications, such as pain, stiffness, and limb length discrepancy, were mentioned in <60% of the consent forms, whilst loosening and wear of the prosthesis affecting the long-term outcomes of the joint replacement was mentioned in 11.5% of the consent forms. This is extremely relevant in patients undergoing joint replacement at a younger age. Minor complications, such as scarring, were documented in <50% consent forms and rare complications, such as death, in 5.5%. Obtaining patient-specific consent for each patient poses several challenges. It influences clinic planning, theatre list planning, and staffing numbers, all of which have their own logistic and economic issues. Technological advances, such as the use of DVDs, Internet portals, and information booklets have helped provide adequate information about joint replacement surgery but have not significantly improved patient understanding [18].

The study has several limitations. The number of patients is relatively small, a power analysis was not carried out and it has focused on patients undergoing only two procedures. To the authors’ knowledge, however, this is the first prospective study examining patient-specific informed consent and that looks at the influence of age and employment status on this process. The operations we have focused on are two of the most performed orthopedic procedures. The results of this study can help form the basis of a patient-specific informed consent process and can be used to optimize the consenting process for patients undergoing other types of surgical procedures.

## Conclusions

Obtaining consent is a patient-specific process. Patient perception of important points that merit discussion can vary with age and employment status. Return to driving is important for all ages, however, as the population ages, the ability to return to activities of daily living becomes an increasingly important discussion point during the consent process.

## Appendices

Annexures 1 and 2

http://www.orthoconsent.com/documents/THR.doc

http://www.orthoconsent.com/documents/TKR.doc

Annexure 3

Informed Consent in Patients Undergoing Primary Hip and Knee Arthroplasty: What Do Patients Want to Know?

Consenting is an important component in the patient care pathway and this study aims to identify risks considered most important and requiring further discussion by yourself before undergoing the procedure as well as to identify sporting and recreational activities you would like to pursue after surgery taking into consideration their values and expectations. Participation in the study is voluntary and the data collected is kept anonymous and confidential.

Hospital No                  _____________

Date of Birth                _____________

Sex                               Male/Female

Occupation                  Retired/Employed

Type of Surgery          Hip Replacement/Knee Replacement

Major complications you would like a further discussion

O Blood Clots

O Pain

O Bleeding

O Prosthesis Wear

O Lifespan of Joint

O Stiffness

O Limb Length Discrepancy

O Infection

Sporting or recreational activity you would like to pursue after surgery

O Driving

O Swimming

O Gardening

O Walking

O Racket Sports

O Hiking

O Yoga

O Dancing

O Golf

O Household Activities

O Stairs

O Walking to the Shop

O Cycling

O Skiing

Other aspects around surgery you would like to discuss?

______________________________________________________________________________________________________________________________________________________________________________________________________________________________________________________
